# Large-scale serosurveillance of COVID-19 in Japan: Acquisition of neutralizing antibodies for Delta but not for Omicron and requirement of booster vaccination to overcome the Omicron’s outbreak

**DOI:** 10.1371/journal.pone.0266270

**Published:** 2022-04-05

**Authors:** Zhenxiao Ren, Mitsuhiro Nishimura, Lidya Handayani Tjan, Koichi Furukawa, Yukiya Kurahashi, Silvia Sutandhio, Kaito Aoki, Natsumi Hasegawa, Jun Arii, Kenichi Uto, Keiji Matsui, Itsuko Sato, Jun Saegusa, Nonoka Godai, Kohei Takeshita, Masaki Yamamoto, Tatsuya Nagashima, Yasuko Mori

**Affiliations:** 1 Division of Clinical Virology, Center for Infectious Diseases, Kobe University Graduate School of Medicine, Kobe, Hyogo, Japan; 2 Department of Clinical Laboratory, Kobe University Hospital, Kobe, Hyogo, Japan; 3 Department of Life Science, Laboratory of Macromolecular Dynamics and X-ray Crystallography, University of Hyogo, Hyogo, Japan; 4 Advanced Photon Technology Division, Life Science Research Infrastructure Group, RIKEN SPring-8 Center, Hyogo, Japan; 5 Hyogo Prefecture Health Promotion Association, Kobe, Hyogo, Japan; University of Hail, SAUDI ARABIA

## Abstract

Continuous appearance of SARS-CoV-2 variants and mass vaccination have been intricately influencing on the COVID-19 situation. To elucidate the current status in Japan, we analyzed totally 2,000 sera in August (n = 1,000) and December (n = 1,000) 2021 collected from individuals who underwent a health check-up. The anti-N seropositive rate were 2.1% and 3.9% in August and December 2021, respectively, demonstrating a Delta variant endemic during that time; it was approximately twofold higher than the rate based on the PCR-based diagnosis. The anti-S seropositive rate was 38.7% in August and it reached 90.8% in December, in concordance with the vaccination rate in Japan. In the December cohort, 78.7% of the sera showed neutralizing activity against the Delta variant, whereas that against the Omicron was much lower at 36.6%. These analyses revealed that effective immunity against the Delta variant was established in December 2021, however, prompt three-dose vaccination is needed to overcome Omicron’s outbreak.

## Introduction

Several turning points have occurred since the COVID-19 pandemic’s emergence in December 2019, and the pandemic has undergone drastic changes with its progress. One of the most important factors is the appearance of the continuous SARS-CoV-2 variants replacing the original variant. In Japan, the Alpha variant (B.1.1.7) replaced the existing strain by around April 2021; the Delta variant (B.1.617.2) then began spreading rapidly throughout the country from July to August 2021(it was called the 5th wave in Japan, S1 Fig) [1–3]. The Omicron variant (B.1.1.529) was first reported in South Africa in November 2021 [4] and has spread worldwide [5] including UK and U.S. (new Omicron infections: >200,000/day in the UK [5] and >1,000,000 Omicron cases per day in the U.S. [6]), and it was expected to invade Japan at the end of 2021. Actually, the spread of Omicron variant has caused largest increase of COVID-19 cases in Japan as of February 2022 (S1 Fig) [1].

The other critical event in the pandemic has been the launch of COVID-19 vaccines. The accelerated development of several vaccine platforms was based on the components of the original SARS-CoV-2 strain as the template, and these vaccines have been demonstrated to be effective for reducing the COVID-19 outbreak [7, 8]. The BNT162b2 mRNA vaccine (Comirnaty^®^, BioNTech-Pfizer, Mainz, Germany/New York, USA) [9] was approved first in Japan and administered to healthcare workers starting in February 2021, then to individuals aged ≥65 old beginning in April 2021, expanding to other populations from May 2021. The mRNA vaccines developed by Moderna [10] were also approved in Japan and have been widely used since May 2021. These vaccines induce immunity against the S antigen, and vaccines based on inactivated virus which include a variety of antigens are not used in Japan. The number of vaccinated individuals in Japan is increasing rapidly, as monitored by the government system [11]; as of December 2021, approx. 74% of the population has received two doses of a vaccine. A three-dose vaccination policy for medical staff was initiated in December 2021, and optional booster vaccination for all adults (≥18 years old) who got the 2nd vaccination over 8 months before has been started in Japan [12].

Periodic seroepidemiologic surveillance is useful to help determine the precise COVID-19 situation [13–17]. We have periodically conducted seroepidemiologic surveillance in one of Japan’s 47 prefectures; i.e., Hyogo prefecture (population 5.4 million) located in the southern-central region of Japan [18]. Trend in the number of COVID-19 cases in Hyogo is well synchronized to that in Japan (S1 Fig), and the age distribution of population in Hyogo is also similar to that of Japan (S1 Table). Our previous seroepidemiologic surveillance conducted in October 2020 revealed that 0.15% of 10,377 sera had neutralizing activity against SARS-CoV-2 infection [18]. In addition to estimate the SARS-CoV-2 infection rate by detecting anti-N antibodies, the vaccination rate is also estimated by separately detecting the anti-S antibodies in this serosurveillance.

In the present study, we conducted seroepidemiologic surveys of COVID-19 in Hyogo prefecture in August and December 2021, which respectively correspond to the beginning and the end of the pandemic’s 5th wave in Japan. We observed that the antibody positivity rate for the N antigen of SARS-CoV-2 increased to 2.1% and 3.9% in those surveys, respectively, indicating the infectious rate of the 5th wave.

## Materials and methods

### Serum samples for seroepidemiologic analysis

The serum samples were collected from individuals who underwent a regular occupational health check-up at the clinics of Hyogo Prefecture Health Promotion Association, Kobe, Japan. Individuals who came to clinics on their own because they feel ill were not included. The 1,000 sera collected during the period from July 19 to August 6, 2021 are referred to herein as the August 2021 cohort. The 1,000 sera collected during the period from November 22 to December 8, 2021 are referred to as the December 2021 cohort. Information of sex and age were collected for the participants, while information of the vaccination status and SARS-CoV-2 infection history were not asked in this study. Exclusion is only for the individuals who declared opt-out.

### Detection of anti-Spike antibodies by ELISA

Anti-Spike antibodies in the human sera were detected by an anti-S enzyme-linked immunosorbent assay (ELISA) as described in our previous study (manuscript submitted). Briefly, each well of a 96-well ELISA plate (Corning, New York, NY) was coated with 100 ng of Spike protein dissolved in a 100 mM carbonate buffer (pH 9.0) and left at 4°C overnight. After a wash with phosphate-buffered saline (PBS) containing 0.1% Tween 20 (PBST), PBST supplemented with 1% bovine serum albumin was added as a blocking buffer, followed by incubation at 4°C for 2 hr. Each serum serially diluted from 1:40 to 1:5120 in the blocking buffer were added to the plate and then incubated at 37°C for 1 hr.

After a wash with PBST, goat anti-human IgG with conjugated horseradish peroxidase (abcam, Cambridge, MA) diluted 1:10000 with PBST was added, followed by incubation at 37°C for 1 hr. After a wash with PBST, ABTS solution (Roche Diagnostics, Indianapolis, IN) was added, and the plate was incubated at room temperature for 40 min in the dark. The reaction was stopped by adding 100 μl of 1.5% (w/v) oxalic acid dehydrate solution.

The optical density at wavelength 405 nm (OD_405_) was measured using the plate reader Multiskan FC (Thermofisher Scientific, Waltham, MA). We validated the anti-S ELISA by using 9 sera from COVID-19 patients, and 12 sera from healthy volunteers (S2 Fig). The details of the COVID-19 sera was provided in S2 Table. Note that five of the sera have been analyzed for their neutralizing antibody titers and reported in our previous study [19], although the anti-S ELISA was performed for the sera for the first time in this study. We set a cut-off value of 0.3 for the 1:40 dilution to define anti-S positivity considering the values for native sera; average 0.131 and standard deviation 0.020. The anti-S ELISA showed apparently high values for COVID-19 sera (S2 Fig), although it does not detect the IgA and IgM.

The value of the area under the curve (AUC) was used to evaluate the anti-S antibody titer [20, 21]. The AUCs were calculated for the plot of OD_405_ values according to dilution factors, and an arbitrary value of 1 was given as the width for a twofold dilution step.

### Detection of anti-N antibody by ECLIA

An electrochemiluminescence immunoassay (ECLIA) was conducted using the cobas e801 module (Roche Diagnostics, Rotkreuz, Switzerland) as in our previous study [18]. The Elecsys Anti-SARS-CoV-2 assay kit (Roche Diagnostics) was used for the detection of antibodies against the SARS-CoV-2 nucleocapsid (N). The measurement of anti-N antibody was performed according to the manufacturer’s instructions, and samples with a cut-off index (COI) >1.0 were diagnosed as positive.

### Viruses

The SARS-CoV-2 Biken-2 (B2) strain, which contains the Spike D614G mutation (here called D614G) was provided by BIKEN Innovative Vaccine Research Alliance Laboratories (Osaka, Japan). The whole genome sequence of the B2 strain has been deposited in the DNA Data Bank of Japan (DDBJ) with the accession number LC644163. The SARS-CoV-2 B.1.167.2 Delta variant (GISAID ID: EPI_ISL_2158617) and the B.1.1.529 Omicron variant (GISAID ID: EPI_ISL_7418017) were provided by Japan’s National Institute of Infectious Disease.

### Neutralization assay

The neutralization activity was measured as described [18, 22] using authentic viruses. Briefly, Vero E6 (TMPRSS2) cells [23] was seeded in 96-well microplate. Heat treated sera (at 56°C for 30 min) were two-fold serially diluted with Dulbecco’s Modified Eagle’s Medium. The diluted sera were mixed with the 100 median tissue culture infectious dose (TCID50) of SARS-CoV-2 variants, incubated at 37°C for one hour, and then added to confluent cells in the 96-well plate. After incubation for six days, the cytopathic effect (CPE) of viruses were monitored by microscopy. The maximum dilution rate of each serum at which no CPE was observed was defined as the neutralizing titer. The sera for which CPE was observed even at the two-fold dilution condition was deemed as negative. The use of SARS-CoV-2 viruses was restricted to the BSL3 Laboratory at the Kobe University.

### Statistical analysis

The Kruskal-Wallis test, Dunn’s multiple comparison test, and Mann-Whitney U-test were performed by GraphPad Prism 8 (GraphPad Software, San Diego, CA). The Spearman’s rank correlation factor was also calculated by the GraphPad Prism 8. The difference of anti-N seropositive rate between August and December cohorts by each age group was statistically analyzed by Fisher’s exact test and χ^2^ test with the *R* software [24]. In all statistical tests, *P*-values <0.05 were considered significant.

### Ethics statement

The seroepidemiologic surveillance was approved by the Ethics Committee of Kobe University Graduate School of Medicine (approval code: B2156702). The Hyogo Prefecture Health Promotion Association was also granted approval for study participation under the same Ethics Committee. Opt-out method was used to get consent of each participant in this study. Information about this retrospective observational study was published on the website of Kobe University Hospital, along with the opportunity to opt out.

To validate the anti-S ELISA, sera from COVID-19 patients and healthy volunteers were used under approval of the Ethical Committee of Kobe University Graduate School of Medicine (approval code B200200), and written informed consents were obtained from the donors.

## Results

### Large-scale seroepidemiologic surveillance of the anti-N positive rate

**Fig 1:**
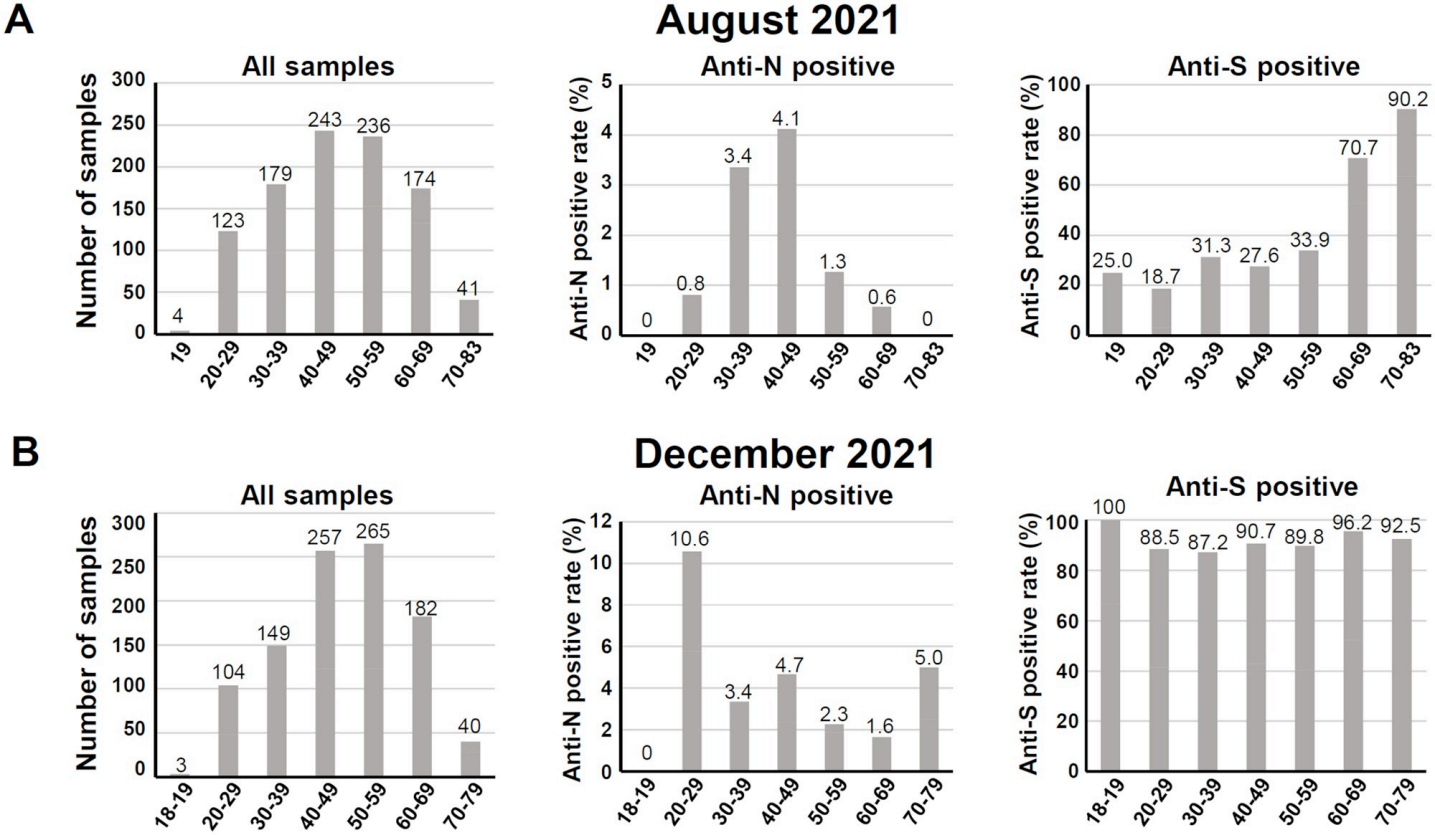
The anti-N and anti-S antibody seroprevalence of the August 2021 and the December 2021 cohorts. **A**: *Left panel*: The age distribution of the August 2021 cohort divided into six age groups: 18–19, 20–29, 30–39, 40–49, 50–59, 60–69, and 70–83 years. *Middle panels*: The anti-N-positive rate and (*right panels*) anti-S-positive rate by the age groups. **B**: The age distribution of the December 2021 cohort: 19, 20–29, 30–39, 40–49, 50–59, 60–69, and 70–79 years.

The results of our large-scale seroepidemiologic surveillance in Hyogo prefecture, Japan performed in August 2021 and December 2021 are as follows. As many as 1,000 samples in each cohort were collected from individuals who underwent a regular occupational health check-up at Hyogo Prefecture Health Promotion Association clinics. The demographic data are summarized in Table 1, and the age distributions are depicted in the left panels of Fig 1A and 1B for the August 2021 and December 2021 cohorts, respectively. These cohorts were composed mainly of persons of 20–69 years old with a smaller number of persons aged 70–80, with few individuals <20 years old and no subjects aged <18 years old, due to the lack of occasion of health check-up for children at the clinic.

**Table 1:** Demographic information of the cohorts for the large-scale seroepidemiologic surveillance.

	August 2021			December 2021		
	All	Male	Female	All	Male	Female
n (%)	1,000 (100%)	587 (58.7%)	413 (41.3%)	1000 (100%)	508 (50.8%)	492 (49.2%)
Age, yrs, median, (range)	48 (19–83)	51 (20–83)	44 (19–78)	49 (18–79)	42 (19–79)	49 (18–75)
Age groups, yrs, n:						
18–19*	4	0	4	3	2	1
20–29	123	41	82	104	48	56
30–39	179	97	82	149	85	64
40–49	243	134	109	257	123	134
50–59	236	153	83	265	123	142
60–69	174	133	41	182	99	83
70–83^†^	41	29	12	40	28	12

* The August 2021 cohort contains only 19 years old in this age group

^†^ The December 2021 cohort contains up to 79 years old in this age group.

The SARS-CoV-2 anti-N antibodies which show past infection by SARS-CoV-2 were detected by the ECLIA [18, 25], and 21 of the 1,000 samples (2.1%) in the August 2021 cohort were deemed positive, whereas 39 of the 1,000 samples (3.9%) in the December 2021 cohort were positive (Table 2). The age distribution of positive cases is summarized in Table 2 and illustrated in the middle panels of Fig 1A and 1B for the August and December 2021 cohorts, respectively. In the August 2021 cohort, the anti-N positive rate for the age groups 30–39 and 40–49 were relatively high at 3.4% (6/179) and 4.1% (10/243), respectively, and no positive cases were observed for the age groups 18–19 and 70–83 years (Fig 1A, middle panel).

**Table 2:** Numbers of anti-N- and anti-S-positive samples by age groups.

	August 2021			December 2021		
	All (n = 1,000)	Male (n = 587)	Female (n = 413)	All (n = 1,000)	Male (n = 508)	Female (n = 492)
**SARS-CoV-2 anti-N antibody (ECLIA)**						
Positive no. by age group, yrs, n						
18–19*	0	0	0	0	0	0
20–29	1	0	1	11	6	5
30–39	6	5	1	5	5	0
40–49	10	7	3	12	6	6
50–59	3	3	0	6	5	1
60–69	1	1	0	3	1	2
70–83^†^	0	0	0	2	1	1
Positive no. in all subjects, n (%)	21 (2.1%)	16 (2.7%)	5 (1.2%)	39 (3.9%)	24 (4.7%)	15 (3.0%)
**SARS-CoV-2 anti-S antibody (ELISA)**						
Positive no. by age group, yrs, n						
18–19*	1	0	1	3	2	1
20–29	23	4	19	92	43	49
30–39	56	30	26	130	74	56
40–49	67	28	39	233	110	123
50–59	80	48	32	238	107	131
60–69	123	92	31	175	95	78
70–83^†^	37	26	11	37	26	11
Positive no. in all subjects, n (%)	387 (38.7%)	228 (38.8%)	159 (38.5%)	908 (90.8%)	457 (90.0%)	449 (91.3%)

* The August 2021 cohort contains only 19 years old in this age group

^†^ The December 2021 cohort contains up to 79 years old in this age group.

Notably, the December 2021 cohort exhibited the noticeable positive rate 10.6% (11/104) for the age group 20–29 (Fig 1B, middle panel), and the difference was significant according to the Fisher’s exact test with the P-value 0.0014 (S3 Table). In contrast to the August 2021 cohort, the oldest age group (i.e., 70–79 yrs) of the December 2021 cohort showed the positive rate 5.0% (2/40), however the difference is not significant by the Fisher’s exact test with the P-value 0.2407.

The positive rate for age groups 30–39 and 40–49 were not largely changed at 3.4% (5/149) and 4.7% (12/257), respectively. These results demonstrate that the infection of the younger (20–29 yrs) and older (70–79 yrs) groups increased during the pandemic’s 5th wave.

### Large-scale seroepidemiologic surveillance of the anti-S positive rate

The anti-S antibodies of the August and December cohorts were analyzed by the anti-S ELISA, which included the vaccination and/or infection history. With the cut-off of 0.3 for the 40-fold serum dilution, the positivity rates were 38.7% (387/1,000 sera) and 90.8% (908/1,000 sera) in the August and the December cohorts, respectively (Table 2). The age distribution of anti-S-positive cases is summarized in Table 2 and shown in the right panels of Fig 1A and 1B. In the August cohort, the anti-S positive rates for the age groups 60–69 and 70–83 were relatively high at 70.7% (123/174) and 90.2% (37/41), respectively (Fig 1A, right panel). The December cohort demonstrated a flattened positive rate at a high level (87.2%–100%) across all of the age groups tested (Fig 1B, right panel).

As expected, majority of the anti-N-positive sera were also anti-S-positive, with one exception for the August cohort and another for the December cohort.

### Quantitative analysis of the anti-S antibody in the two cohorts

**Fig 2:**
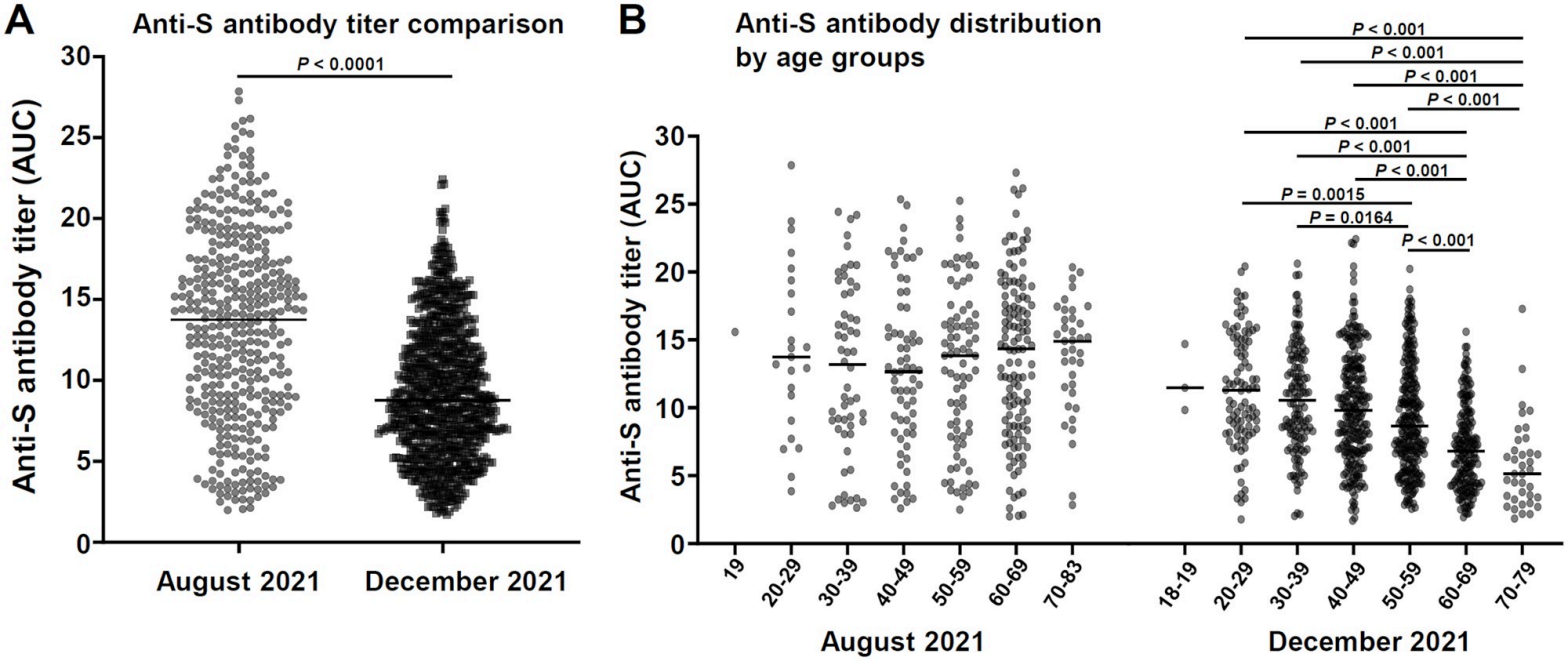
Quantitative evaluation of the anti-S antibody amount by the anti-S ELISA. **A**: The distributions of the anti-S ELISA AUC values were compared between the August 2021 and December 2021 cohorts. *P-*value, Mann-Whitney U-test. *Black bars*: medians. **B**: The distributions in panel A plotted separately for the age groups. The Kruskal-Wallis test was performed for the multiple comparison among age groups in each of the August and December cohort. The *P-*values were obtained by the subsequent Dunn’s multiple comparison test among age groups for significant data (<0.05) separately in each cohort. *Black bars*: medians.

We quantitatively analyzed the anti-S positive sera in the August 2021 and December 2021 cohorts to estimate and compare the cohorts’ anti-S antibody levels. The quantitative distributions of the anti-S antibody titer are shown in Fig 2A. The August 2021 cohort’s distribution showed a significantly higher level of AUCs with the median 13.8 compared to that of the December 2021 cohort with the median 8.8 (Mann-Whitney U-test).

We further analyzed the distributions of the anti-S antibody titer by separating the age groups (Fig 2B). There was no significant difference in the distribution among age groups in the August cohort by the Kruskal-Wallis test, whereas the distribution in the December cohort showed significant differences among the age groups, showing a clear tendency of decreased anti-S antibody levels in the older age group (70–79), indicating that the lower anti-S antibody titer did affect infection.

### Neutralization activity of the anti-S-positive sera from the August 2021 cohort

**Fig 3:**
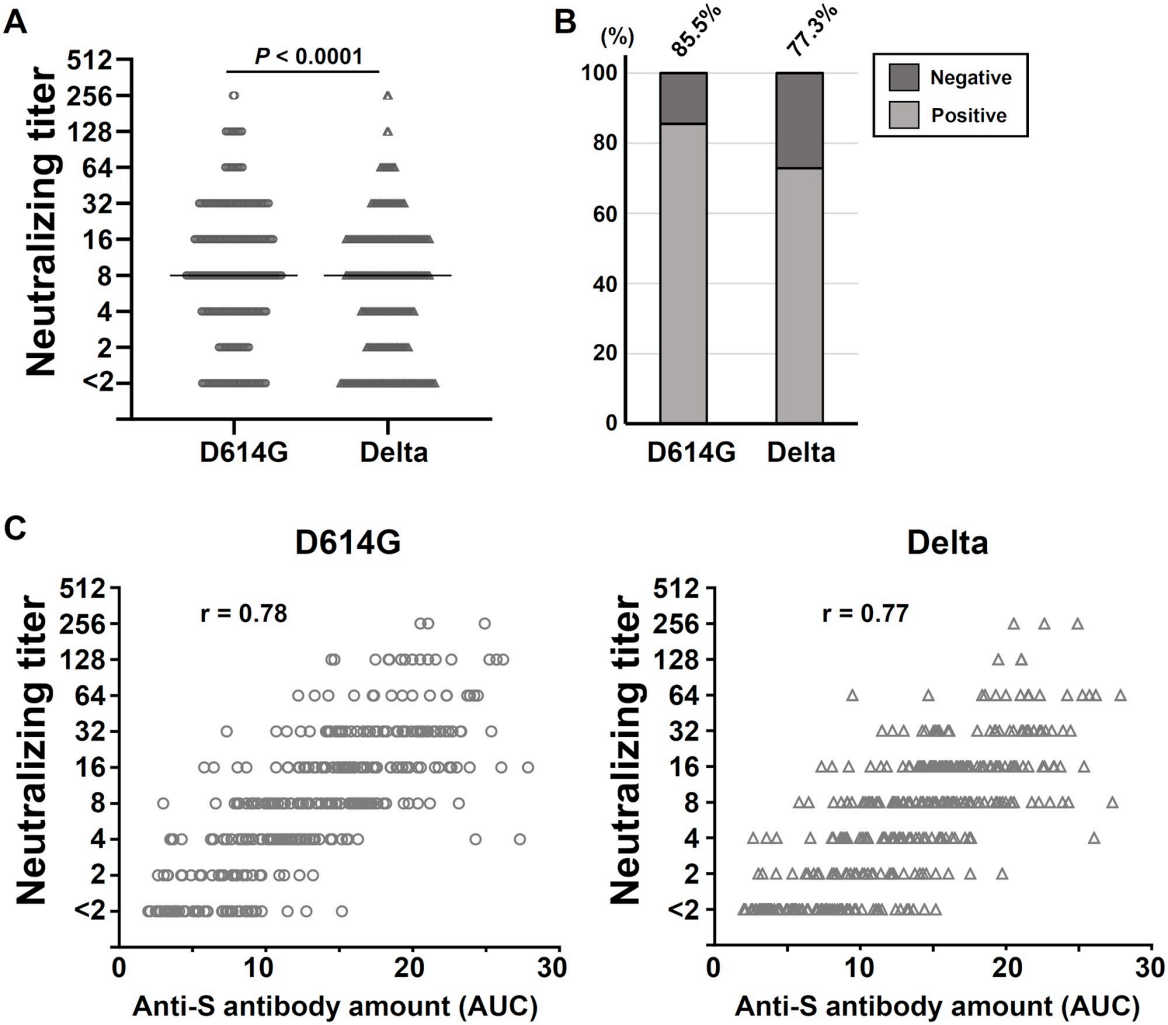
**A**: Neutralization antibody titers of the anti-S-positive subset of the August 2021 cohort plotted for D614G and the Delta variant. *Black bars*: medians. *P-*value, Mann-Whitney U-test. **B**: The neutralization-positive rates for D614G and the Delta variant were evaluated from the data shown in panel A, with the titers <2 deemed negative.

To analyze the relationship between the anti-S antibody and neutralizing activity against SARS-CoV-2, the neutralization antibody titers of the anti-S-positive subset (n = 387) from the August 2021 cohort were quantitatively evaluated. Fig 3A depicts the distributions of neutralization antibody titers against D614G and the Delta variant. Although the distributions appeared similar with the same median value, i.e., 8, the Mann-Whitney U-test result indicated that the neutralizing titers for D614G were significantly higher than those for the Delta variant. The neutralization positivity rate in the anti-S-positive subset also showed a similar result in that there were slightly higher rates for D614G and Delta at 85.5% and 77.3%, respectively (Fig 3B), indicating the efficacy of the two-dose vaccine for the Delta variant as well. The correlation plot between the anti-S AUC and the neutralizing antibody titer showed the positive correlation with the Spearman’s rank correlation factor 0.78 and 0.77 for SARS-CoV-2 D614G and Delta, respectively (Fig 3C).

### Neutralizing activities of the sera collected in December 2021 against the Delta and Omicron variants

**Fig 4:**
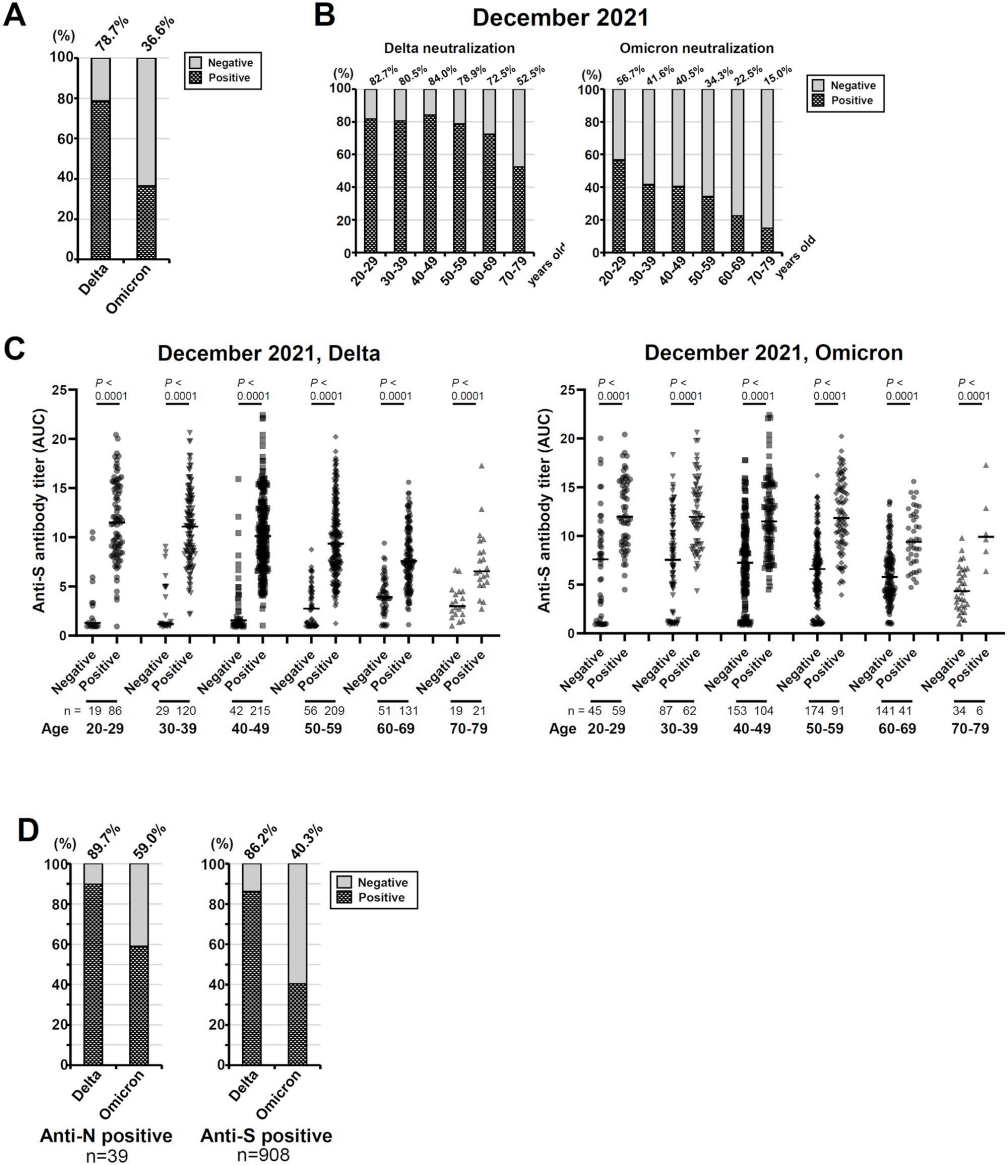
Neutralization activity of the sera collected in December 2021 against the Delta and Omicron variants. **A**: The neutralization-positive rates of the entire December 2021 cohort (n = 1,000) plotted for the Delta and Omicron variants. **B**: The neutralization-positive rates for the Delta and Omicron variants by age groups. The data in panel A were reanalyzed as in Fig 2B. **C**: Comparison of the anti-S antibody amount between the neutralization-positive and -negative groups. The complete set of 1,000 sera of the December 2021 cohort were subgrouped according to result of the neutralization assay against the Delta variant and the Omicron variant, and the anti-S antibody titers were plotted. Statistical analysis was performed separately for each age group in each cohort one by one, comparing the neutralizing positive and negative groups by Mann-Whitney U-test with showing the *P*-value. *Black bars*: medians. D: The neutralization-positive rate calculated for the anti-N-positive (n = 39) and anti-S (n = 908) subsets obtained by extracting the data from those shown in panel A.

Detailed analysis of the December 2021 cohort is especially important because it represents the status after the Delta pandemic and before the Omicron pandemic (S1 Fig). Thus, we analyzed the neutralization positivity rate against the Delta and Omicron variants in all 1,000 sera of the December 2021 cohort. The neutralization positivity rate against the Delta variant was 78.7% (787/1,000) in the December 2021 cohort, implying effective immunity was conferred to residents (Fig 4A, Table 3). In contrast, the neutralization positivity rate against the Omicron variant was quite low at 36.6% (366/1,000) (Fig 4A, Table 3), implying the vulnerability of the cohort against infection by the Omicron variant.

**Table 3:** Neutralization positive rate of the sera collected on December 2021.

	Neutralization positive number, (%)	
	Delta	Omicron
by age groups, yrs,		
18–19 (n = 3)	3 (100%)	3 (100%)
20–29 (n = 104)	86 (82.7%)	59 (56.7%)
30–39 (n = 149)	120 (80.5%)	62 (41.6%)
40–49 (n = 257)	216 (84.0%)	104 (40.5%)
50–59 (n = 265)	209 (78.9%)	91 (34.3%)
60–69 (n = 182)	132 (72.5%)	41 (22.5%)
70–79 (n = 40)	21 (52.5%)	6 (15.0%)
in all subjects (n = 1,000)	787 (78.7%)	366 (36.6%)

A further analysis of the same data with a separation of age groups revealed that the neutralization positivity rate against the Delta variant tended to be lower in older age groups (Fig 4B, Table 3). The same trend was also observed in the neutralization positivity rate against the Omicron variant (Fig 4B, Table 3), and in the oldest age group (70–79 yrs) only 15.0% of the sera demonstrated the neutralization activity.

The comparison of the anti-S antibody titers between the neutralization-positive and -negative sera revealed that the neutralization-positive group had clearly higher titers of anti-S antibody in both the Delta and Omicron cases (Fig 4C), supporting our speculation that the neutralization activity of the sera was attributed to the anti-S antibodies.

Lastly, we focused on the anti-N-positive (n = 39) and anti-S positive (n = 908) subsets of the December 2021 cohort. The neutralization-positive rate against the Delta and the Omicron variants for the anti-N positive subset were 89.7% and 59.0%, respectively (Fig 4D). These values were slightly higher than those for the entire set of 1,000 sera or those for the anti-N negative subset (78.3% and 35.7% for the Delta and the Omicron variants, respectively), indicating superior immune responses in the individuals with a history of SARS-CoV-2 infection. The neutralization positive rate for the anti-S positive subset were 86.2% and 40.3% against the Delta and the Omicron variants, respectively.

## Discussion

In combination with other methods used to understanding the spread of infectious diseases such as the polymerase chain reaction (PCR)-based diagnosis and antigen tests, periodic cross-sectional seroepidemiologic surveillance is a powerful approach. Our present surveillance in Japan’s Hyogo prefecture revealed that the anti-N-positive rate, which represents the SARS-CoV-2 infection rate, was 2.1% in the August 2021 cohort and 3.9% in the December 2021 cohort. In our October 2020 serosurveillance [18], the anti-N-positive rate by the same ECLIA method for 1,000 sera from the same Hyogo Prefecture Health Promotion Association clinics was 0.4%. The increase in the anti-N-positive rate in Hyogo prefecture was well synchronized with the cumulative number of the COVID-19 cases based on PCR results (S3 Fig). The increased rate of anti-N seroprevalence was 0.17% per month from October 2020 to August 2021 and 0.45% per month from August 2021 to December 2021. The steep increase during the latter period is accounted for by the spread of the Delta variant in Japan’s so-called ’5th wave’ (S1 Fig). Serosurveillance by Japanese government at the five areas in Japan, that is Tokyo, Osaka, Miyagi, Aichi, and Fukuoka, during 14-25^th^, 2020 also used the Roche ECLIA as our study, in combination with Abbott chemiluminescent immunoassay (CLIA) (available from: https://www.mhlw.go.jp/content/000734482.pdf, Japanese). The result calculated for the ECLIA only to compare with our data was well consistent with our surveillance (S3 Fig) at that time point, although we need to wait for the next surveillance to compare with our data obtained before and after the 5^th^ wave.

We suspected that the SARS-CoV-2 infection rate estimated by the seroprevalence of anti-N antibody would be higher than the rate indicated by the PCR-based diagnoses due to the existence of asymptomatically or mildly infected individuals whose cases may not have been detected since they never underwent testing by PCR analysis or antigen test. The anti-N ECLIA used here is a high sensitivity method widely detecting IgG, IgA, and IgM, it has advantage for the use of serosurveillance [26]. Indeed, the anti-N ECLIA-based infection rates revealed in the present analyses, i.e., 2.1% and 3.9% for August and December 2021, were respectively higher than the rates 0.84% (46,381 cases/5,465 thousand people in Hyogo prefecture as of August 6, 2021) and 1.4% (78,898 cases/5,465 thousand people in Hyogo prefecture as of December 8, 2021) estimated from the reported cases based on PCR diagnoses [1]. The fold difference was nearly constant for both periods, reflecting the synchronized increases in the anti-N-positive rate and the reported cases (S3 Fig), i.e., 2.5- to 2.6-fold lower for the PCR-based estimation.

Our cohorts were mainly composed of 20- to 80-years-olds, and it may contribute to the higher infection rate of our serosurveillance compared to the PCR-based report which covers the all age distribution. Since the infection rate are not equal for each age group (S4 Table), we should estimate the effect of the biased sampling. The PCR-based infection rate by age groups in Hyogo prefecture and Japan were summarized in S4 Table according to a Japanese government report, and we calculated the expected positive case number per 1,000 samples for each age group in the Hyogo Prefecture at the time points. If we assumed to collect samples from each age groups in the same biased distribution as our cohorts, we expected the PCR positive rate become 0.94% (from 0.85% reported) and 1.6% (from 1.4% reported) at the time points of August and December 2021, respectively. Thus, we suggest that our biased sample collection may have only slight effect on the result of infection rate. The difference between the PCR-based infection and serosurveillance is consistent with the report that a substantial portion of SARS-CoV-2-infected individuals are asymptomatic [27], leading to the inaccuracy of surveillance rates determined solely by PCR diagnosis.

Two-dose COVID-19 vaccination has been intensively promoted in Japan from May to December 2021, and approx. 74% population has finished the two-dose vaccination (as of January 6, 2022; [11]). This is a relatively high rate considering the global average (46%) in January 2022 (January 6, 2022; https://covid19.who.int/table). Our anti-S seroprevalence result for the August 2021 cohort (38.7%) represents the build-up period of immunity for residents (Table 2, Fig 1A), and it is well consistent with Hyogo prefecture’s single and two-dose vaccination rates at 32.79% and 42.05%, respectively as of August 6, 2021. In December 2021, when the vaccination rate had reached a plateau in Japan, our survey revealed an extremely high seropositive rate, i.e., 90.8% (Table 2, Fig 1B). This result implied that the vaccinations in Japan (*i*) have conferred immunity to residents, and (*ii*) may have contributed to the current COVID-19 situation having calmed down in December 2021 (S1 and S3 Figs).

The anti-S seroprevalence observed in the present study is clearly higher compared to the reported single- and two-dose vaccination rates in Hyogo prefecture as of December 8, 2021 at 73.09% and 74.01%, respectively. This difference might be accounted for by the bias in age groups of the present cohorts which contained very few people <20 years old, whose vaccination rate has been relatively low compared to those of other age groups (https://web.pref.hyogo.lg.jp/kf16/coronavaccine.html#wakuchinsessyujyoukyou). Indeed, the rate of two-dose vaccination completion was 79.4–83.1%, 88.5–91.2%, and 94.3–98.2% for 20–49, 50–69, and >70 years old, respectively according to a Japanese government report (Available from: https://www.kantei.go.jp/jp/content/nenreikaikyubetsu-vaccination_data.xlsx). In addition, our cohorts consisted of individuals who underwent a health check-up, and this could have contributed to a biased selection of socially active and health-conscious people.

Our comparison of the anti-N and anti-S seropositive rates among the age groups revealed differing tendencies between the August 2021 and December 2021 cohorts (Fig 1A and 1B). The August 2021 cohort—which was enrolled during a period of vaccination progression and the beginning of the Delta variant’s rampage—represents competing effects of the vaccinations and the Delta variant. The high anti-S-positive rate for the age groups 60–69 and 70–83 years observed herein is explained by the priority vaccination for elderly individuals (>65 yrs) in Japan (Fig 1A). Interestingly, the high anti-S-positive rate might be related to the relatively low infection rate (low anti-N-positive rate) in the present study’s elderly age groups (Fig 1A), indicating the vaccines’ efficacy.

In contrast, the December 2021 cohort was enrolled during the period in which the ’fully vaccinated’ rate had reached a plateau and the increase in the number of COVID-19 cases due to the Delta variant had dropped to a low level (S1 Fig). The anti-N-positive rate was increased in the 20–29 and 70–79 age groups in this cohort (Fig 1B). Although the anti-S-positive rate of the December 2021 cohort showed no apparent difference among age groups (Fig 1B), our quantitative analysis of the anti-S antibody titers suggested that the increased infection for the 70- to 79-year-olds was the result of the decreased anti-S antibody titers for the elderly age groups (Fig 2B). Indeed, the anti-S antibody level was significantly lower for the December 2021 cohort compared to that for the August 2021 cohort (Fig 2A), reflecting the decline of acquired immunity after vaccination (Furukawa et al. [18] manuscript submitted) [28, 29]. Regarding the increase in the positive rate of anti-N antibody in the 20–29 age group, we speculate that this increase might reflect increased social activity among these younger individuals.

As shown in Fig 4A, the neutralization assay using the SARS-CoV-2 viruses revealed that the December 2021 cohort had a high neutralization-positive rate for the Delta variant, i.e., 78.7%. This result demonstrated the two-dose vaccination suppressed the spread of the Delta variant, leading to a transient convergence of the COVID-19, resulting in the relatively low infection rate of 3.9%.

The emergence of the Omicron variant is a key event worldwide, as is the spread of the Delta variant. As of December 2021, European countries and U.S. have been invaded by the Omicron variant, which has also been detected across Japan. Actually, the Omicron variant has caused a largest increase of COVID-19 cases ever so called “6^th^ wave” in Japan (S1 Fig). Our neutralization assay against the Omicron variant for the December 2021 cohort, which represents the current Japanese vaccinated population, demonstrated a quite low positive rate, 36.6% (Fig 4A) which appears to be due at least in part to several mutations that occurred in the S protein [5, 30, 31]. We thus contend that the three-dose vaccination to boost the immunity against Omicron is needed to prevent and suppress the further spread of the Omicron variant. Sera with neutralizing activity against the Omicron variant in our December 2021 cohort showed trend of higher anti-S IgG amount (Fig 4C). Indeed, it has been demonstrated by our group (Furukawa et al. [18] manuscript submitted) and other groups [5, 31] that the third dose booster vaccination is effective to raise neutralization titers against the Omicron variant. Our present findings also demonstrate the efficacy of vaccines for SARS-CoV-2 suppression. The booster should be required, in order to increase the population’s immunity to the novel variant Omicron.

## Supporting information

S1 Fig The COVID-19 situation in Japan (nationwide) and in Hyogo prefecture. The daily COVID-19 cases reported based on PCR diagnoses in Japan (A) and in Hyogo prefecture (B) are plotted from January 2020 to February 2022. The data were obtained from the website provided by Japan’s Ministry of Health, Labour and Welfare (https://www.mhlw.go.jp/stf/covid-19/open-data_english.html) [1] and modified. The surges of SARS-CoV-2 spread that have occurred in Japan, called the 1st to 6th waves, are indicated in panel A. In the panel (B), the time points of our serosurveillance in this study were indicated by dashed lines.
(DOCX)

S2 Fig Anti-S ELISA validation using sera from healthy volunteers and COVID-19 patients. The OD405 values for the 40-fold serum dilution was plotted. Background of the sera from COVID-19 patients were shown in S2 Table. Black bars: medians.
(DOCX)

S3 Fig The COVID-19 situation in Hyogo prefecture and Japan. The cumulative infection cases reported based on the PCR diagnosis [1] are plotted as the solid line, and the infection rates obtained by our serosurveillance surveys are plotted as the dashed line. The anti-N-positive rate shown by the ECLIA for the October 2020 cohort was obtained from our previous study [18]. The rapid increase in the number of COVID-19 cases, i.e., the so-called 2nd to 5th waves in Japan, are also indicated. For comparison, the ECLIA-based infection rate calculated from the data of Japanese government serosurveillance (https://www.mhlw.go.jp/content/000734482.pdf, Japanese) was also indicated.
(DOCX)

S1 Table Demographic information of Hyogo prefecture and Japan and bias in age distribution in our cohorts. (DOCX)

S2 Table Anti-S ELISA for sera from COVID-19 patient. (DOCX)

S3 Table Statistical analysis on the infection rate between August and December by age groups. (DOCX)

S4 Table Reported PCR-based infection rate by age groups for Hyogo prefecture and Japan. (DOCX)

## References

[1] Trend in the number of newly confirmed cases (daily) in Japan. https://covid19.mhlw.go.jp/public/opendata/confirmed_cases_cumulative_daily.csv. Accessed 1 March 2022.

[2] Murayama H, Kayano T, Nishiura H. Estimating COVID-19 cases infected with the variant alpha (VOC 202012/01): an analysis of screening data in Tokyo, January-March 2021. Theor Biol Med Model 2021; 18:13. doi: 10.1186/s12976-021-00146-x 34273991 PMC8286039

[3] Ito K, Piantham C, Nishiura H. Predicted dominance of variant Delta of SARS-CoV-2 before Tokyo Olympic Games, Japan, July 2021. Euro Surveill 2021; 26. 10.2807/1560-7917.ES.2021.26.27.2100570 PMC8268651 34240695

[4] Omicron variant: what you need to know. https://www.cdc.gov/coronavirus/2019-ncov/variants/omicron-variant.html. Accessed December 15 2021.

[5] Dejnirattisai W, Huo J, Zhou D, et al. Omicron-B.1.1.529 leads to widespread escape from neutralizing antibody responses. bioRxiv 2021. 10.1016/j.cell.2021.12.046 PMC8723827 35081335

[6] COVID-19 Weekly Epidemiological Update Edition 70, published 14 December 2021. https://www.who.int/docs/default-source/coronaviruse/situation-reports/20211214_weekly_epi_update_70.pdf. Accessed 13 January 2022 2022.

[7] Lopez Bernal J, Andrews N, Gower C, et al. Effectiveness of the Pfizer-BioNTech and Oxford-AstraZeneca vaccines on covid-19 related symptoms, hospital admissions, and mortality in older adults in England: test negative case-control study. BMJ 2021; 373:n1088. doi: 10.1136/bmj.n1088 33985964 PMC8116636

[8] Izda V, Jeffries MA, Sawalha AH. COVID-19: A review of therapeutic strategies and vaccine candidates. Clin Immunol 2021; 222:108634. doi: 10.1016/j.clim.2020.108634 33217545 PMC7670907

[9] Polack FP, Thomas SJ, Kitchin N, et al. Safety and Efficacy of the BNT162b2 mRNA Covid-19 Vaccine. N Engl J Med 2020; 383:2603–15. doi: 10.1056/NEJMoa2034577 33301246 PMC7745181

[10] Baden LR, El Sahly HM, Essink B, et al. Efficacy and Safety of the mRNA-1273 SARS-CoV-2 Vaccine. N Engl J Med 2021; 384:403–16. doi: 10.1056/NEJMoa2035389 33378609 PMC7787219

[11] Open data of vaccination in Japan. https://cio.go.jp/c19vaccine_dashboard. Accessed 1 March 2022.

[12] COVID-19 Vaccine Booster Shots (3rd Dose). https://www.mhlw.go.jp/stf/covid-19/booster.html. Accessed 1 March 2022.

[13] Zurcher K, Mugglin C, Suter-Riniker F, et al. Seroprevalence of SARS-CoV-2 in healthcare workers from outpatient facilities and retirement or nursing homes in a Swiss canton. Swiss Med Wkly 2021; 151. doi: 10.4414/SMW.2021.w30021 34495604

[14] Santosh CS, Tukaram DP, Maharudra K, et al. Seroprevalence of SARS-CoV-2 Antibodies and Associated Factors in Health Care Workers. J Assoc Physicians India 2021; 69:11–2. 34472805

[15] Batool H, Chughtai O, Khan MD, Chughtai AS, Ashraf S, Khan MJ. Seroprevalence of COVID-19 IgG antibodies among healthcare workers of Pakistan: a cross-sectional study assessing exposure to COVID-19 and identification of high-risk subgroups. BMJ Open 2021; 11:e046276. doi: 10.1136/bmjopen-2020-046276 34400447 PMC8370836

[16] Smigelskas K, Petrikonis K, Kasiulevicius V, et al. SARS-CoV-2 Seroprevalence in Lithuania: Results of National Population Survey. Acta Med Litu 2021; 28:48–58. doi: 10.15388/Amed.2020.28.1.2 34393628 PMC8311832

[17] Naesens R, Mertes H, Clukers J, et al. SARS-CoV-2 seroprevalence survey among health care providers in a Belgian public multiple-site hospital. Epidemiol Infect 2021; 149:e172. doi: 10.1017/S0950268821001497 34372955 PMC8365049

[18] Furukawa K, Arii J, Nishimura M, et al. Seroepidemiological Survey of the Antibody for Severe Acute Respiratory Syndrome Coronavirus 2 with Neutralizing Activity at Hospitals: A Cross-sectional Study in Hyogo Prefecture, Japan. Jma J 2021; 4:41–9. doi: 10.31662/jmaj.2020-0094 33575502 PMC7872787

[19] Kurahashi Y, Sutandhio S, Furukawa K, et al. Cross-Neutralizing Breadth and Longevity Against SARS-CoV-2 Variants After Infections. Front Immunol 2022. doi: 10.3389/fimmu.2022.773652 35281007 PMC8907139

[20] Amanat F, Stadlbauer D, Strohmeier S, et al. A serological assay to detect SARS-CoV-2 seroconversion in humans. Nat Med 2020; 26:1033–6. doi: 10.1038/s41591-020-0913-5 32398876 PMC8183627

[21] Gaebler C, Wang Z, Lorenzi JCC, et al. Evolution of antibody immunity to SARS-CoV-2. Nature 2021; 591:639–44. doi: 10.1038/s41586-021-03207-w 33461210 PMC8221082

[22] Tjan LH, Nagano T, Furukawa K, et al. The Neutralizing Antibody Response against Severe Acute Respiratory Syndrome Coronavirus 2 and the Cytokine/Chemokine Release in Patients with Different Levels of Coronavirus Diseases 2019 Severity: Cytokine Storm Still Persists Despite Viral Disappearance in Critical Patients. Jma J 2021; 4:1–7. doi: 10.31662/jmaj.2020-0083 33575497 PMC7872782

[23] Matsuyama S, Nao N, Shirato K, et al. Enhanced isolation of SARS-CoV-2 by TMPRSS2-expressing cells. Proc Natl Acad Sci U S A 2020; 117:7001–3. doi: 10.1073/pnas.2002589117 32165541 PMC7132130

[24] Team RC. R: A language and environment for statistical computing. Vienna, Austria.: R Foundation for Statistical Computing, 2016.

[25] Egger M, Bundschuh C, Wiesinger K, et al. Comparison of the Elecsys(R) Anti-SARS-CoV-2 immunoassay with the EDI enzyme linked immunosorbent assays for the detection of SARS-CoV-2 antibodies in human plasma. Clin Chim Acta 2020; 509:18–21. doi: 10.1016/j.cca.2020.05.049 32485155 PMC7261064

[26] Stone M, Grebe E, Sulaeman H, et al. Evaluation of Commercially Available High-Throughput SARS-CoV-2 Serologic Assays for Serosurveillance and Related Applications. Emerg Infect Dis 2022; 28:672–83. doi: 10.3201/eid2803.211885 35202525 PMC8888213

[27] Alene M, Yismaw L, Assemie MA, et al. Magnitude of asymptomatic COVID-19 cases throughout the course of infection: A systematic review and meta-analysis. PLoS One 2021; 16:e0249090. doi: 10.1371/journal.pone.0249090 33755688 PMC7987199

[28] Altawalah H. Antibody Responses to Natural SARS-CoV-2 Infection or after COVID-19 Vaccination. Vaccines (Basel) 2021; 9. doi: 10.3390/vaccines9080910 34452035 PMC8402626

[29] Tre-Hardy M, Cupaiolo R, Wilmet A, et al. Immunogenicity of mRNA-1273 COVID vaccine after 6 months surveillance in health care workers; a third dose is necessary. J Infect 2021; 83:559–64. doi: 10.1016/j.jinf.2021.08.031 34437927 PMC8380546

[30] Wang L, Cheng G. Sequence analysis of the emerging SARS-CoV-2 variant Omicron in South Africa. J Med Virol 2021. doi: 10.1002/jmv.27516 34897752

[31] Liu L, Iketani S, Guo Y, et al. Striking Antibody Evasion Manifested by the Omicron Variant of SARS-CoV-2. Nature 2021. doi: 10.1038/s41586-021-04388-0 35016198

