# Supplementary material for: Large-scale serosurveillance of COVID-19 in Japan: Acquisition of neutralizing antibodies for Delta but not for Omicron and requirement of booster vaccination to overcome the Omicron’s outbreak
Source: PLoS One. 2022 Apr 5;17(4):e0266270. doi: 10.1371/journal.pone.0266270 (PMC8982849; doi:10.1371/journal.pone.0266270)
Supplement: S1 Fig — The daily COVID-19 cases reported based on PCR diagnoses in Japan (A) and in Hyogo prefecture (B) are plotted from January 2020 to February 2022. The data were obtained from the website provided by Japan’s Ministry of Health, Labour and Welfare (https://www.mhlw.go.jp/stf/covid-19/open-data_english.html) [1] and modified. The surges of SARS-CoV-2 spread that have occurred in Japan, called the 1st to 6th waves, are indicated in panel A. In the panel (B), the time points of our serosurveillance in this study were indicated by dashed lines. (DOCX) [file pone.0266270.s001.docx]

**
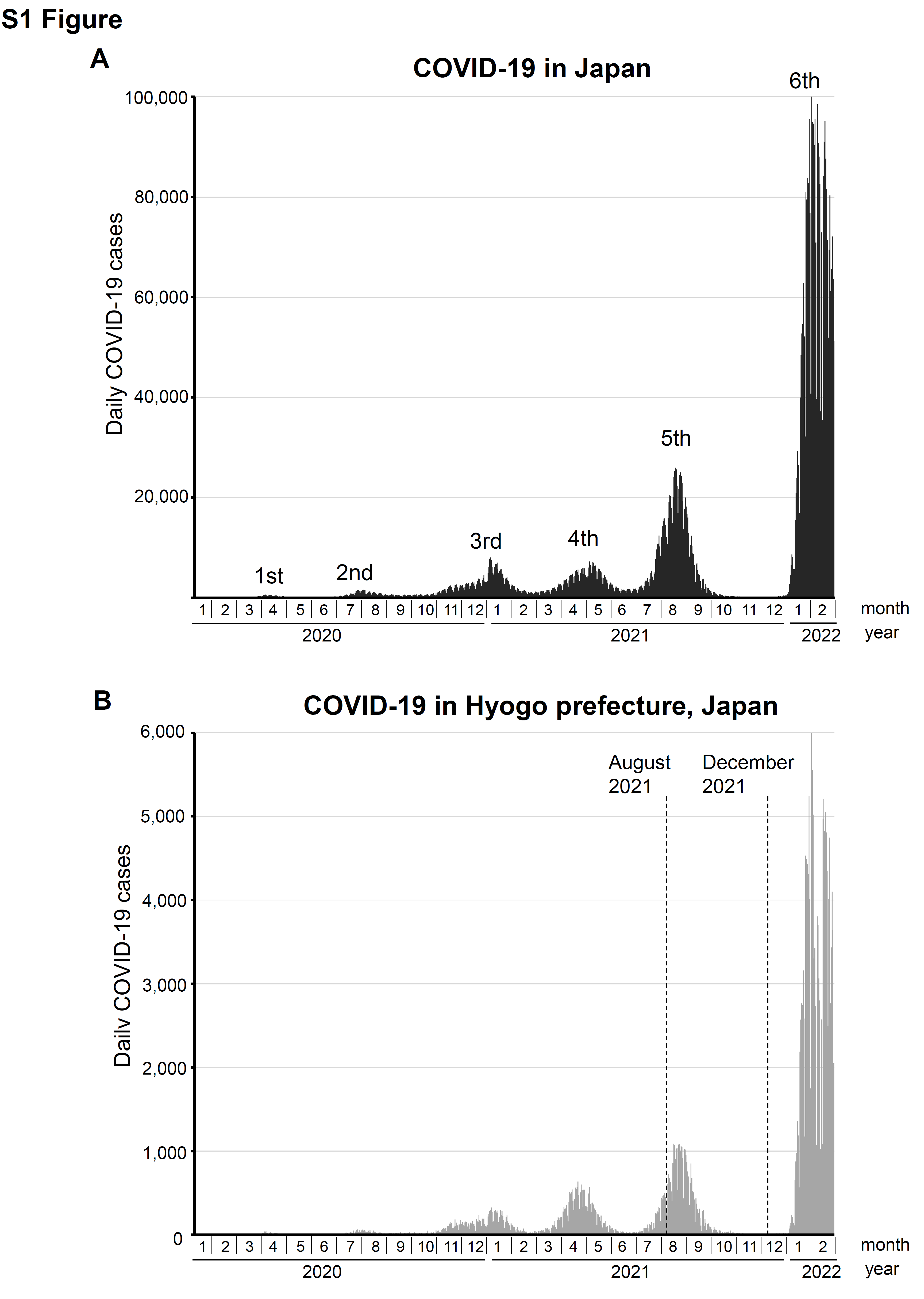
**

**S1 Fig.** The COVID-19 situation in Japan (nationwide) and in Hyogo prefecture. The daily COVID-19 cases reported based on PCR diagnoses in Japan (A) and in Hyogo prefecture (B) are plotted from January 2020 to February 2022. The data were obtained from the website provided by Japan's Ministry of Health, Labour and Welfare (https://www.mhlw.go.jp/stf/covid-19/open-data_english.html) [1] and modified. The surges of SARS-CoV-2 spread that have occurred in Japan, called the 1st to 6th waves, are indicated in panel A. In the panel (B), the time points of our serosurveillance in this study were indicated by dashed lines.
