# Supplementary material for: Large-scale serosurveillance of COVID-19 in Japan: Acquisition of neutralizing antibodies for Delta but not for Omicron and requirement of booster vaccination to overcome the Omicron’s outbreak
Source: PLoS One. 2022 Apr 5;17(4):e0266270. doi: 10.1371/journal.pone.0266270 (PMC8982849; doi:10.1371/journal.pone.0266270)
Supplement: S2 Fig — The OD405 values for the 40-fold serum dilution was plotted. Background of the sera from COVID-19 patients were shown in S2 Table. Black bars: medians. (DOCX) [file pone.0266270.s002.docx]

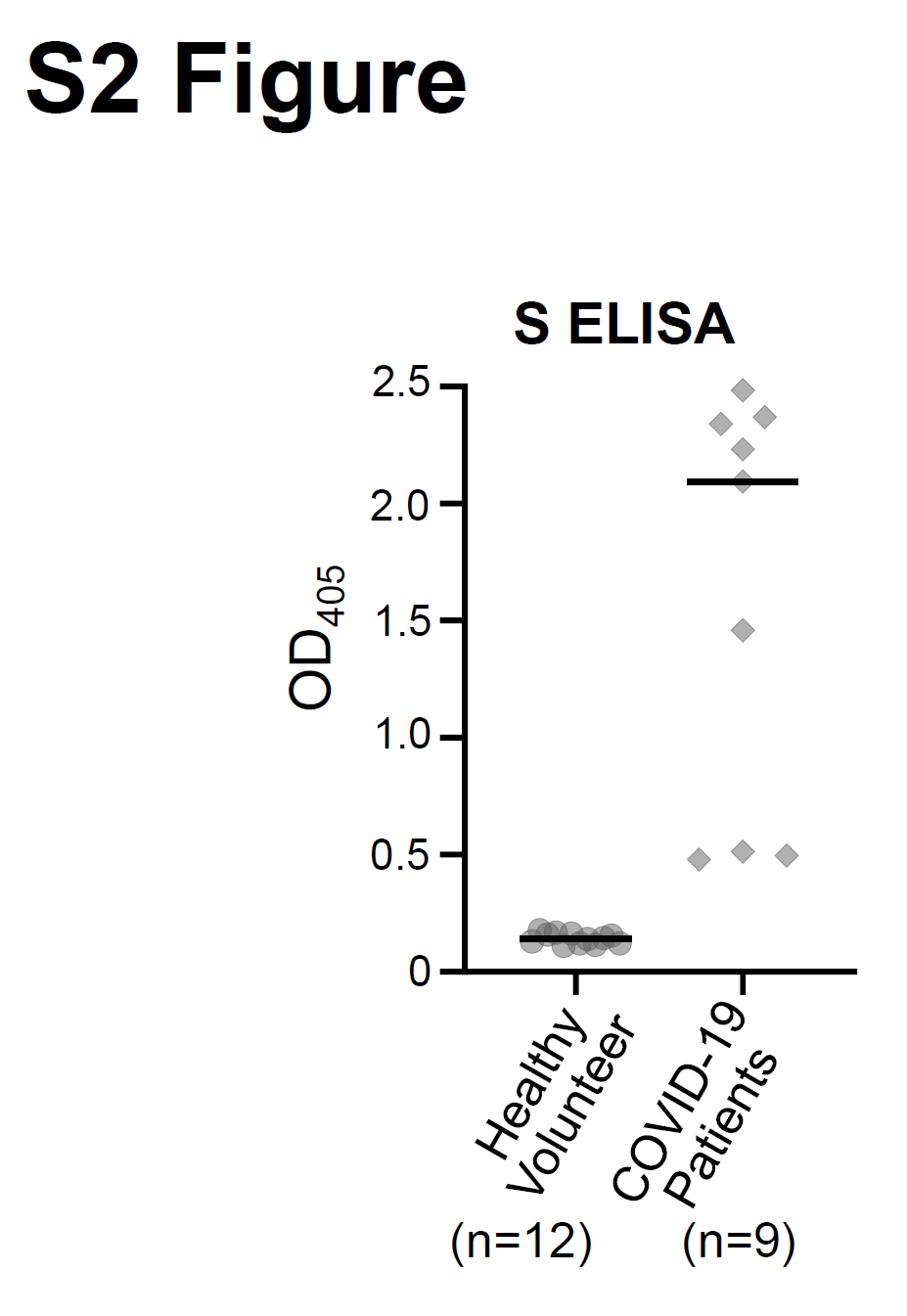


**S2 Fig.** Anti-S ELISA validation using sera from healthy volunteers and COVID-19 patients. The OD405 values for the 40-fold serum dilution was plotted. Background of the sera from COVID-19 patients were shown in S2 Table. *Black bars:* medians.
