# Supplementary material for: Large-scale serosurveillance of COVID-19 in Japan: Acquisition of neutralizing antibodies for Delta but not for Omicron and requirement of booster vaccination to overcome the Omicron’s outbreak
Source: PLoS One. 2022 Apr 5;17(4):e0266270. doi: 10.1371/journal.pone.0266270 (PMC8982849; doi:10.1371/journal.pone.0266270)
Supplement: S3 Fig — The cumulative infection cases reported based on the PCR diagnosis [1] are plotted as the solid line, and the infection rates obtained by our serosurveillance surveys are plotted as the dashed line. The anti-N-positive rate shown by the ECLIA for the October 2020 cohort was obtained from our previous study [18]. The rapid increase in the number of COVID-19 cases, i.e., the so-called 2nd to 5th waves in Japan, are also indicated. For comparison, the ECLIA-based infection rate calculated from the data of Japanese government serosurveillance (https://www.mhlw.go.jp/content/000734482.pdf, Japanese) was also indicated. (DOCX) [file pone.0266270.s003.docx]

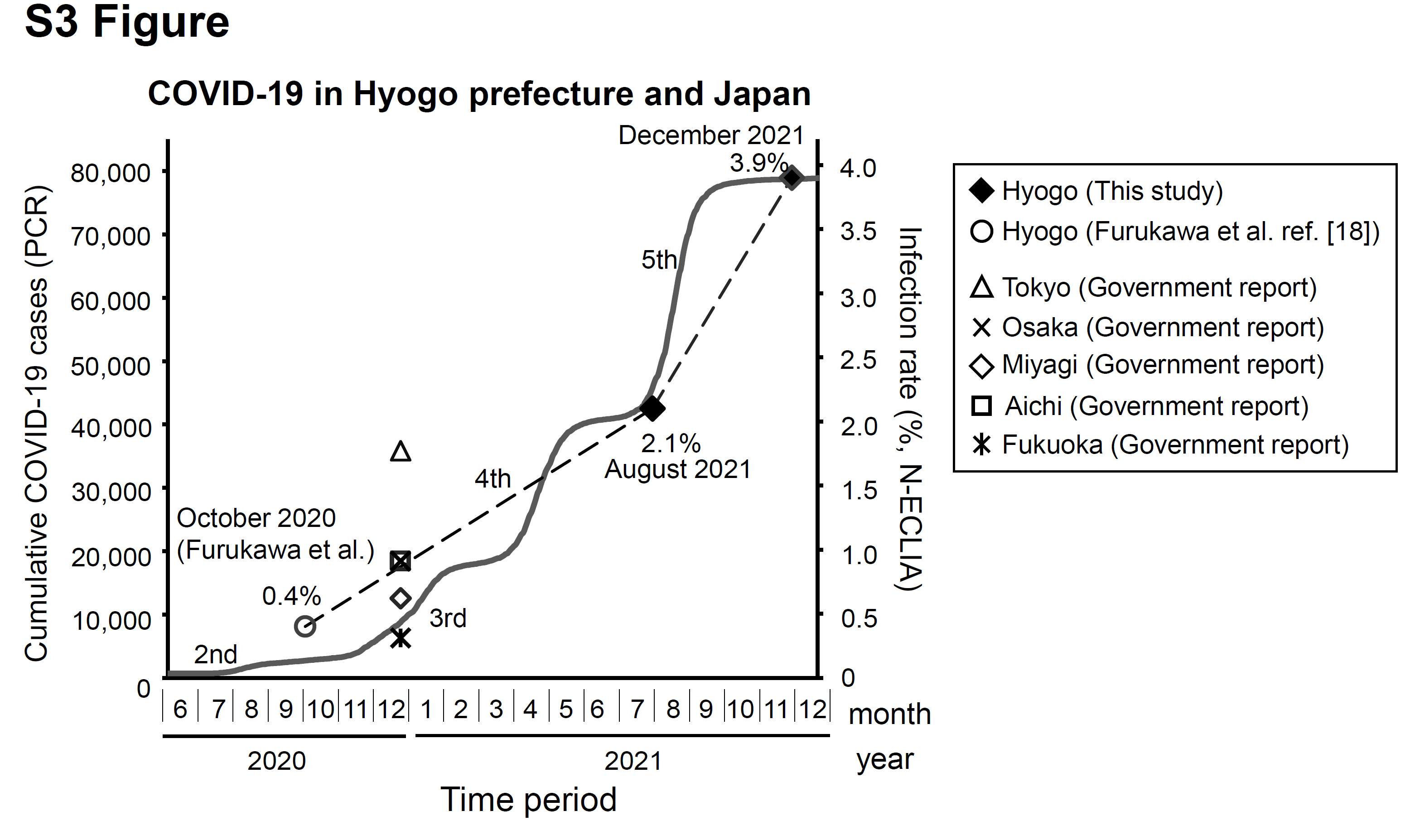


**S3 Fig.** **The COVID-19 situation in Hyogo prefecture and Japan.** The cumulative infection cases reported based on the PCR diagnosis [1] are plotted as the solid line, and the infection rates obtained by our serosurveillance surveys are plotted as the dashed line. The anti-N-positive rate shown by the ECLIA for the October 2020 cohort was obtained from our previous study [18]. The rapid increase in the number of COVID-19 cases, i.e., the so-called 2nd to 5th waves in Japan, are also indicated. For comparison, the ECLIA-based infection rate calculated from the data of Japanese government serosurveillance (https://www.mhlw.go.jp/content/000734482.pdf, Japanese) was also indicated.
