# Supplementary material for: Large-scale serosurveillance of COVID-19 in Japan: Acquisition of neutralizing antibodies for Delta but not for Omicron and requirement of booster vaccination to overcome the Omicron’s outbreak
Source: PLoS One. 2022 Apr 5;17(4):e0266270. doi: 10.1371/journal.pone.0266270 (PMC8982849; doi:10.1371/journal.pone.0266270)
Supplement: S1 Table — (DOCX) [file pone.0266270.s004.docx]

## S1 Table. Demographic information of Hyogo prefecture and Japan and bias in age distribution in our cohorts.

|  | **Population, thousand (% ratio of all)**  **(1^st^ October 2021)** | | **Sample numbers in this study^‡^**  **(% ratio of all)** | |
| --- | --- | --- | --- | --- |
| **Age groups, yrs** | **Hyogo Prefecture^*^** | **Japan^†^** | **August 2021** | **December 2021** |
| 0-9 | 426 (7.8%) | 9,657 (7.7%) | 0 (0%) | 0 (0%) |
| 10-19 | 495 (9.1%) | 11,082 (8.8%) | 4 (0.4%) | 3 (0.3%) |
| 20-29 | 515 (9.4%) | 12,703 (10.1%) | 123 (12.3%) | 104 (10.4%) |
| 30-39 | 579 (10.6%) | 14,213 (11.3%) | 179 (17.9%) | 149 (14.9%) |
| 40-49 | 791 (14.5%) | 18,345 (14.5%) | 243 (24.3%) | 257 (25.7%) |
| 50-59 | 737 (13.5%) | 16,677 (13.2%) | 236 (23.6%) | 265 (26.5%) |
| 60-69 | 677 (12.4%) | 15,678 (12.4%) | 174 (17.4%) | 182 (18.2%) |
| 70-79 | 740 (13.5%) | 16,254 (12.9%) | 39 (3.9%) | 40 (4.0%) |
| 80-89 | 403 (7.4%) | 9,146 (7.3%) | 2 (0.2%) | 0 (0%) |
| >90 | 102 (1.9%) | 2,391 (1.9%) | 0 (0%) | 0 (0%) |
| all | 5,465 (100%) | 126,146 (100%) | 1,000 (100%) | 1,000 (100%) |

* Data was based on a Hyogo prefecture office report for the time point of 1^st^ Oct, 2020: https://web.pref.hyogo.lg.jp/kk11/jinkou-tochitoukei/jinkoubunseki.html (Accessed on 28 February)

^†^Data was based on the government report for the time point of 1^st^ Oct, 2020: https://www.e-stat.go.jp/en/stat-search/files?page=1&layout=datalist&toukei=00200524&tstat=000000090001&cycle=7&year=20200&month=0&tclass1=000001011679&tclass2val=0　(Accessed on 28 February)

^‡^The data shown in Table 1 were also shown here to compare the age distribution of this study and general population.
