# Supplementary material for: Large-scale serosurveillance of COVID-19 in Japan: Acquisition of neutralizing antibodies for Delta but not for Omicron and requirement of booster vaccination to overcome the Omicron’s outbreak
Source: PLoS One. 2022 Apr 5;17(4):e0266270. doi: 10.1371/journal.pone.0266270 (PMC8982849; doi:10.1371/journal.pone.0266270)
Supplement: S2 Table — (DOCX) [file pone.0266270.s005.docx]

## S2 Table. Anti-S ELISA for sera from COVID-19 patient.

| **Patient ID** | **Sex** | **Age** | **Severity** | **Days post onset** | **anti-S ELISA OD_405_** | **Reference^*^** |
| --- | --- | --- | --- | --- | --- | --- |
| P1 | M | 77 | Severe | 10 | 0.481 | - |
| P2 | F | 25 | Mild | 205 | 0.496 | Kurahashi et al. [19] |
| P3 | M | 58 | critical | 14 | 2.094 | - |
|  |  |  |  | 21 | 2.340 | - |
| P4 | M | 79 | severe | 13 | 2.485 | - |
| P5 | M | 44 | Mild | 38 | 0.513 | Kurahashi et al. [19] |
| P6 | F | 49 | severe | 114 | 2.371 | Kurahashi et al. [19] |
| P7 | M | 69 | moderate | 105 | 2.232 | Kurahashi et al. [19] |
| P8 | F | 52 | Mild | 47 | 1.458 | Kurahashi et al. [19] |

*Note that five of the sera have been analyzed for their neutralizing antibody titers and reported in our previous study [19], although the anti-S ELISA was performed for the sera for the first time in this study.
