# Supplementary material for: Large-scale serosurveillance of COVID-19 in Japan: Acquisition of neutralizing antibodies for Delta but not for Omicron and requirement of booster vaccination to overcome the Omicron’s outbreak
Source: PLoS One. 2022 Apr 5;17(4):e0266270. doi: 10.1371/journal.pone.0266270 (PMC8982849; doi:10.1371/journal.pone.0266270)
Supplement: S3 Table — (DOCX) [file pone.0266270.s006.docx]

## S3 Table. Statistical analysis on the infection rate between August and December by age groups.

|  | **August** | | **December** | |  |
| --- | --- | --- | --- | --- | --- |
| **Age groups, yrs** | **Positive rate** | **95% confidence interval** | **Positive rate** | **95% confidence interval** | **Difference between**  **August and December, *P*-value** |
| 18-19 | 0% | 0.0-60.2% | 0% | 0-70.8% | 1.0000^†^ |
| 20-29 | 0.8% | 0.0-4.5% | 10.6% | 5.4-18.1% | 0.0014^†^ |
| 30-39 | 3.4% | 1.2-7.2% | 3.4% | 1.1-7.7% | 1.0000^‡^ |
| 40-49 | 4.1% | 2.0-7.4% | 4.7% | 2.4-8.0% | 0.8295^‡^ |
| 50-59 | 1.3% | 0.3-3.7% | 2.3% | 0.84-4.9%) | 0.5105^†^ |
| 60-69 | 0.6% | 0.0-3.2% | 1.6% | 0.34-4.7% | 0.6233^†^ |
| 70-83^§^ | 0% | 0-8.6% | 5.0% | 0.61-16.9% | 0.2497^†^ |

^*^ The 95 percent confidence intervals were obtained by the binominal exact test.

^†^ *P-*value was obtained by Fisher’s exact test, considering small positive numbers.

^‡^ *P-*value was obtained by χ^2^ test.

^§^ The December 2021 cohort contains up to 79 years old in this age group
