# Supplementary material for: Large-scale serosurveillance of COVID-19 in Japan: Acquisition of neutralizing antibodies for Delta but not for Omicron and requirement of booster vaccination to overcome the Omicron’s outbreak
Source: PLoS One. 2022 Apr 5;17(4):e0266270. doi: 10.1371/journal.pone.0266270 (PMC8982849; doi:10.1371/journal.pone.0266270)
Supplement: S4 Table — (DOCX) [file pone.0266270.s007.docx]

## S4 Table. Reported PCR-based infection rate by age groups for Hyogo Prefecture and Japan.

|  | **Ratio of PCR-based cases for the age groups at the timepoint, %***  **(Expected positive case number for 1,000 samples at the time point) ^†^** | | | |
| --- | --- | --- | --- | --- |
|  | **as of 10^th^ August, 2021** | | **as of 7^th^ December, 2021** | |
| Age groups, yrs | Hyogo Prefecture | Japan | Hyogo Prefecture | Japan |
| 0-9 | 3.5% (3.8) | 3.9% (4.0) | 5.4% (10.0) | 5.5% (9.8) |
| 0-19 | 8.8% (8.2) | 8.6% (7.8) | 11.1% (17.7) | 10.4% (16.2) |
| 20-29 | 18.2% (16.4) | 24.3% (19.1) | 19.4% (29.7) | 24.9% (33.8) |
| 30-39 | 12.3% (9.9) | 16.0% (11.3) | 13.2% (18.0) | 16.5% (20.0) |
| 40-49 | 14.6% (8.6) | 15.3% (8.3) | 15.1% (15.1) | 15.7% (14.7) |
| 50-59 | 14.7% (9.3) | 13.0% (7.8) | 13.9% (14.9) | 12.3% (12.7) |
| 60-69 | 9.3% (6.4) | 7.3% (4.7) | 7.7% (9.0) | 6.0% (6.6) |
| 70-79 | 9.1% (5.7) | 5.9% (3.6) | 7.2% (7.7) | 4.5% (4.8) |
| 80-89 | 6.9% (7.9) | 4.1% (4.5) | 5.1% (10.0) | 3.1% (5.8) |
| >90 | 2.6% (11.8) | 1.6% (6.7) | 1.8% (13.9) | 1.1% (7.9) |
| all | 100% (8.5) | 100% (7.9) | 100% (14.4) | 100% (13.7) |

* This ratio was calculated as follows:

$$\frac{case number of the age group}{case number of all} \times100$$

The case number for each age group was obtained from a government report (https://covid19.mhlw.go.jp/public/opendata/newly_confirmed_cases_detail_weekly.csv). Although the data were available only from September 2^nd^ 2020, the case number before that is likely ignorable considering the low number at the duration as shown in S1 Fig.

^†^ This number means expected positive case if we collect 1,000 sample for the age group at the area. It was calculated based on PCR-based report as follows:

$$\frac{\left( PCR based cases for all \right)\times\left( ratio of cases for the age group \right)}{population for the age group} \times1,000$$
